# Observations on the Belladonna

**Published:** 1800-07

**Authors:** Thomas Wainwright

**Affiliations:** Member of the Royal College of Surgeons in London


### *Obſervations on the Belladonna.*

By THOMAS WAINWRIGHT, Member of the Royal College of Surgeons in London.

To the EDITORS of the MEDICAL and PHYSICAL JOURNAL.

Gentlemen,

THE account publiſhed in your Journal for April, page 367, of the effects of the belladona applied to the eyes, previous to their being operated on for the cataract, induces me to trouble you with a few obſervations on the medical uſe of this plant.

Ray has related a caſe, where, from the application of the green leaf of the belladonna to the eye, the iris became ſo affected, that the pupil would not contract even in the brighteſt light. The leaf being removed, the eye was gradually reſtored to its former ſtate; and this was repeated three ſeparate times. It does not ſeem to have been known, that the dilatation of the pupil and paralytic ſtate of the iris, invariably followed the free application of the belladonna to the eye.

Twelve

Twelve years ago I became acquainted with this fact, from accidental and perfonal experience; and in 1791, I mentioned this remarkable effect of the belladonna to the Phyfical Society of Guy's, and at the fame time ftated my expectation, that advantage would arife from its application in certain difeafes of the eye. I have been in the habit of applying the juice and the extract of the belladonna diluted with water, very freely, in many cafes of inflammation of the eye for feveral years. In almoft every inftance where the belladonna has been brought in contact with the cornea, the pupil gradually dilates, and the iris becomes lefs and lefs fenfible to the ftimulus of light. The degree of dilatation can very much be regulated at pleafure, by repeating the application; in fome inftances, the iris has almoft difappeared, and temporary lofs of vifion enfued: the ufe of the belladonna collyrium has been continued through one and two weeks. When the belladonna is difcontinued, the eye in every inftance is reftored to its natural ftate; the iris recovers its fenfibility in about twenty-four or forty-eight hours. The application of the belladonna has lefs effect on the eyes of fome; and in feveral difeafes of the eye, the pupil is not fo readily dilated by it. The ufe of the belladonna does not in any way weaken or injure the fight; on the contrary, I have thought that a moderate artificial dilatation has, in my own cafe, rendered my vifion ftronger and more fteady; and that the glare of fun-fhine has been lefs offenfive to me. There is no doubt on my mind, but that the external ufe will be found greatly to facilitate the operations for the cataract. The belladonna collyrium is ferviceable in acute inflammations of the eye, and in inflammations arifing from external violence; in the fcrofulous opthalmia it has, with me, had no good effect; and it has alfo failed in an opthalmia, which afterwards turned out to be venereal. Specks, attended with confiderable inflammation, were in two inftances removed, by repeatedly infinuating the extract of the belladonna under the eye-lids: the expreffed juice diluted with water, is more effectual than a folution of the extract in water. I have thought the belladonna lotion more efficacious in cafes where the pupil is to be quickly dilated from its ufe. The beauty of the berries of this plant, has frequently led children to eat them in confiderable quantities; and in feveral inftances, twelve and even twenty-four hours have elapfed before any medicine has been exhibited, and the berries have continued to be voided by ftool during two and three days; yet, I have not heard of any cafe which has terminated fatally:—the berries do not appear to be fo deleterious as the leaves. From a very free application of the diluted extract of the belladonna to an eryfipelas of the leg, leg, attended with excoriations, the pupil became dilated, with irritability of the iris, and temporary blindneſs, with pain of the head, convulſive twitchings, and that peculiar idiotic derangement of the mind, which ariſes from the belladonna having been taken into the ſtomach in improper quantities.

The belladonna has been given internally, joined with calomel and tartar emetic, in obſtinate caſes of chronic rheumatiſm, in certain cutaneous eruptions, and in caſes of ulcers and of tumours threatening to become cancerous, with good effect. Temporary amendment has followed its uſe in cancer; half a grain of the extract is a full doſe, and ſhould be repeated night and morning, or thrice a day, till the pupil is ſomewhat dilated: the dilatation of the pupil is an excellent criterion of a full doſe. The medicine ſhould not be puſhed further, but this effect be kept up. It is a great error to give medicine in too large doſes; and it has been well obſerved, that medicines, given in very large doſes, often loſe their peculiar effects, and act as mere ſtimulants. I am,

Gentlemen,

Your very obliged humble ſervant,

*Dudley, June* 14, 1800. T. WAINWRIGHT.

