# Errata

**Published:** 1800-08

**Authors:** 


ERRATA.

No. XVII. Page 6, line 10 from the bottom, dele *to be*, and read "*where the pupil is quickly dilated.*"

Page 7, line 2, for *irritability*, read *immobility*.

